# Fluoride-Mediated Immune Damage Through Cytokine Network Regulation of Tregs

**DOI:** 10.3390/toxics13020095

**Published:** 2025-01-26

**Authors:** Bingshu Liu, Siqi Zhu, Qiong Zhang, Fengyu Xie, Dan Wei, Guiyu Fu, Liu Yang, Yanhui Gao, Wei Wei

**Affiliations:** 1 Center for Endemic Disease Control, Chinese Center for Disease Control and Prevention, Harbin Medical University, Harbin 150081, China; liubingshu@yeah.net (B.L.); zhusiqi@hrbmu.edu.cn (S.Z.); 18829414372@163.com (Q.Z.); 2022020100@hrbmu.edu.cn (F.X.); wd7905003@163.com (D.W.); yangliu_1987@163.com (L.Y.); 2 Key Lab of Etiology and Epidemiology, Education Bureau of Heilongjiang Province & Ministry of Health (23618504), Harbin Medical University, Harbin 150081, China; 3 Heilongjiang Provincial Key Lab of Trace Elements, Human Health Harbin Medical University, Harbin 150081, China; 4 Jining Center For Disease Control And Prevention, Shandong Province, Jining 272000, China; fuguiyu1618@163.com

**Keywords:** fluorosis, inflammation, cytokines, regulatory T cells

## Abstract

Long-term fluoride exposure can induce inflammatory responses in various tissues of the body, thereby affecting the inflammatory microenvironment. To explore how fluoride induces changes in immune function within this microenvironment, this study collected baseline information and biological samples from participants in areas with the drinking water type of fluorosis, and simultaneously established Wistar rat models with a 12-week and 24-week fluoride exposure, as well as a 12-week fluoride exposure followed by 12-week pure water feeding regimen. Luminex multiplex assays and enzyme-linked immunosorbent assays (ELISAs) were used to measure cytokine expression levels. Subsequently, correlation analysis, multiple linear regression, and mediation analysis were employed to explore the long-term effects induced by the complex cytokine network during fluoride exposure. The population survey results indicated that fluoride suppressed the expression of pro-inflammatory factors such as Interleukin-2 (IL-2), Interleukin-12 (IL-12), Interferon-γ (IFN-γ), Tumor necrosis factor-α (TNF-α), and anti-inflammatory factors such as Interleukin-4 (IL-4), Interleukin-13 (IL-13), and Interleukin-37 (IL-37), while promoting an increase in the proportion of regulatory T cells (Tregs) in peripheral blood. Among these, IL-2 and IFN-γ mediated the fluoride-induced peripheral Tregs expansion. Animal experiments indicate that the proportion of Tregs in peripheral blood and immune organs increases in a time-dependent manner with fluoride exposure. After reducing the fluoride concentration in the drinking water of rats, the number of Tregs remained significantly elevated. The changes in Treg numbers in the 12-week fluoride feeding group, 24-week fluoride feeding group, and 12-week fluoride feeding followed by 12-week water improvement group were related to the cytokine levels. Therefore, the impact of fluoride on the immune homeostasis has cumulative and long-term effects, and may be related to the accumulation and migration of Tregs induced by fluoride in an inflammatory environment, mediated by cytokines.

## 1. Introduction

Fluoride mainly exists in minerals or sedimentary rocks, entering groundwater through water-rock interactions. Industrial and agricultural growth have further caused fluoride pollution via untreated wastewater and residues [1,2,3]. Over 200 million people worldwide face a systemic homeostasis imbalance and organ dysfunction due to fluoride-contaminated drinking water [4,5,6,7,8,9]. The World Health Organization (WHO) has established a fluoride limit of 1.5 mg/L in drinking water [10], and nations worldwide have since taken measures to lower fluoride concentrations [11]. In China, a 10-year groundwater quality survey found that 15% of the country’s groundwater has a fluoride concentration exceeding 1.0 mg/L [12]. According to the World Health Organization’s statistics on global drinking water quality regulations and standards, 102 countries and regions have set limits for fluoride ion concentrations in water. Among them, 77 countries follow the WHO’s recommended limit, while 8 countries or regions exceed the standard, with the highest concentration reaching 4.0 mg/L [13]. In addition, there are other types of fluorosis in China, including tea-based and coal-burning fluorosis [14,15]. Due to China’s vast geographic distribution, large population, and complex exposure patterns, the national standard limits fluoride concentrations in large-scale centralized water supplies to 1.0 mg/L, and in small-scale centralized water supplies to 1.2 mg/L [14,16]. Fluoride exposure is known to cause damage to multiple organs. In recent years, increasing attention has been given to its detrimental effects on the immune system [5,17].

The immune system is the body’s first line of defense against disease. Numerous studies have shown that the prolonged exposure to high doses of fluoride induces structural abnormalities in lymphoid organs [18,19,20,21,22], promotes immune cell apoptosis, inhibits lymphocyte proliferation, and disrupts the lymphocyte cycle [19,23,24], resulting in immune system dysfunction. Our previous research found that fluoride has immunotoxicity in Wistar rats [25], and later found that moderate or low fluoride exposure can cause immune system damage and induce changes in the number of regulatory T cells (Tregs) in peripheral blood [22]. Although the effects of fluoride on immune cells and immune-active substances have garnered attention and preliminary confirmation from researchers [20,26,27,28], direct evidence of fluoride’s regulation of Tregs in an inflammatory environment is still lacking.

Tregs play a central role in immune regulation by exerting their suppressive functions. In this process, cytokines directly or indirectly contribute to immune-mediated tissue dysfunction [29] and tightly regulate pro-inflammatory and anti-inflammatory responses mediated by immune cells [30]. During inflammation, Tregs are rapidly recruited from the peripheral Tregs pool, while resident Tregs in tissues quickly expand and contribute to tissue repair and inflammation resolution [31,32]. The elevated expression of inflammatory factors is the immune system’s primary response to tissue injury [33]. Previous studies have shown that fluoride can increase the expression levels of inflammatory factors such as Interleukin-1β (IL-1β) and Tumor necrosis factor-α (TNF-α) [29,34]. Therefore, is the accumulation of inflammatory factors induced by fluoride a key factor in the imbalance of Tregs numbers?

This study aims to investigate whether fluoride disrupts the expression of cytokines, thereby disturbing the homeostasis of Tregs numbers. Through a cross-sectional study of residents in low to moderate fluoride exposure areas in China, and animal models with different fluoride exposure durations and doses, we explore the regulatory role of cytokines in Tregs changes under environmental fluoride exposure.

## 2. Materials and Methods

### 2.1. Selection of Study Area and Population

The total number of participants was 375. According to the inclusion and exclusion criteria, a total of 327 adults aged 18 and above were included in this survey. They have all lived in the endemic fluorosis areas of Jishan County, Shanxi Province, China, for more than 5 years. The water fluoride levels in the selected villages were as follows: Xiadi Village: 0.89 mg/L, Jiandong Village: 0.96 mg/L, Shangfei Village: 2.23 mg/L, and Xiafei Village: 2.66 mg/L. After the participants signed the informed consent form, urine and blood samples were collected, and their basic information was gathered. Participants also completed a questionnaire and underwent a physical examination. Participants with incomplete data, hemolyzed blood samples, renal disease, diabetes, immune-related disorders, or a history of or plans for organ transplantation, or those taking immunosuppressive drugs were excluded.

### 2.2. Construction of a Rat Model of Fluorosis via Drinking Water

For this study, 150 male Wistar rats, aged 3 weeks, were purchased from VITALON Laboratory Animal Technology Co., Ltd. (Beijing Vital River Laboratory Animal Technology Co., Ltd., Qualification Number: SCXK (Beijing, China) 2012-0001, 2016-0006, and 2016-0011). All rats were housed in a controlled environment with a constant temperature of 20 ± 2 °C, humidity of 50 ± 15%, and a light-dark cycle, with ad libitum access to food and water. Based on the body surface area of humans and animals, and considering the metabolism and absorption of fluoride in rats, according to calculations, the WHO’s safety threshold for fluoride intake from drinking water (1.5 mg/L) corresponds to a fluoride concentration of 10 mg/L in the drinking water of rats. After 1 week of acclimatization, the 150 rats were randomly assigned to 5 groups (*n* = 30) and provided with drinking water containing 0, 10, 25, 50, or 100 mg/L of fluoride. Although 50 and 100 mg/L are not equivalent to the doses humans are exposed to in natural environments, they are commonly used in animal models of fluorosis and have been widely demonstrated to be robust in rat models of fluorosis [35,36,37]. According to the exposure mode and time of fluoride, it can be divided into three modes: fluoride treatment for 12 weeks (12 w), fluoride treatment for 24 weeks (24 w), and fluoride treatment for 12 weeks and 12 weeks of improve water(12 w12 wi) (Table S1). Rats were euthanized with isoflurane anesthesia at the end of the breeding period.

### 2.3. Preparation of Peripheral Blood Mononuclear Cells (PBMCs)

Then, 2 mL of human peripheral blood or 4 mL of peripheral blood from fluoride-exposed rats, collected in EDTA anticoagulant tubes, was slowly mixed with extraction reagent. The procedure was then strictly followed according to the manufacturer’s instructions (Human: Ficoll-Paque PREMIUM 1.073, Cytiva, GE Healthcare, Chicago, IL, USA and Rat: Ficoll-Paque PREMIUM 1.084, Cytiva, GE Healthcare, Chicago, IL, USA). All experiments were conducted at room temperature.

### 2.4. Preparation of Thymic Single-Cell Suspension

After euthanizing the rats, a portion of the thymus was placed in RPMI-1640 medium containing 1% penicillin-streptomycin. The tissue was then gently torn using sterile forceps. The suspension was then centrifuged (1500 rpm, 10 min) to obtain a thymic single-cell suspension. All experiments were conducted at 4 °C.

### 2.5. Preparation of Spleen Single-Cell Suspension

After euthanizing the rats, a portion of the spleen was placed in RPMI-1640 medium containing 1% penicillin-streptomycin and then gently ground with a pair of sterile slides. The cell suspension was filtered through a 70 μm cell strainer and then centrifuged (1500 rpm, 10 min). Red blood cells were removed using lysis buffer according to the manufacturer’s instructions, yielding a spleen single-cell suspension. All procedures were performed at 4 °C.

### 2.6. Measurement of Urinary Fluoride

Urine samples from both participants and rats were collected and stored at −80 °C for later use. A 1 mL urine sample was diluted five times, and the fluoride concentration was measured using the fluoride ion-selective electrode method [38], according to the “Fluoride Ion Selective Electrode Method” (WS/T 89–2015) of the People’s Republic of China health industry standards. After reading the potential value, calculate the fluoride ion concentration in urine based on c = 10^(E − a)/b^. In the formula, E is the potential value, measured in millivolts (mV); A is the intercept of the standard curve; B is the slope of the standard curve regression equation; and C is the urinary fluoride concentration. All chemicals used in the experiment were of analytical grade, and deionized water was used throughout the procedure.

### 2.7. Measurement of the Proportion of Tregs in the Samples

The antibodies for flow cytometry, including CD3 (Human: 300406, Rat: 2014003), CD4 (Human: 300528, Rat: 201520), and CD25 (Human: 356110, Rat: 202114), were purchased from BioLegend(San Diego, CA, USA). A total of 1 × 10^7^ cells/mL were placed in a 1.5 mL EP tube. The antibody was diluted according to the manufacturer’s instructions, and the cell suspension was incubated at 4 °C in the dark for 30 min. The cells were then centrifuged (3500 rpm, 5 min), resuspended in staining buffer, and analyzed using a BD C6 Plus flow cytometer. Data analysis was performed using FlowJo 10.8.1.

### 2.8. Measurement of Cytokine Expression in the Samples

After diluting 500 µL of serum samples from participants by 1.5 times, cytokine expression levels of IL-1β, Interleukin-2 (IL-2), Interleukin-4 (IL-4), Interleukin-12 (IL-12), Interleukin- 13 (IL-13), Interferon-γ (IFN-γ), and TNF-α were measured using the Bio-Plex Pro Reagent Kit IIIwith Flat Bottom Plate (171304090M, Bio-Plex, Hercules, CA, USA). After diluting 500 µL of rat serum by 5 times, cytokine expression levels of IL-1β, IL-2, IL-4, Interleukin-10 (IL-10), IL-12, IL-13, IFN-γ, Human macrophage inflammatory protein 3α (MIP-3α, CCL20), and TNF-α were measured using the Bio-Plex Pro Rat Cytokine 23-Plex (12005641, Bio-Plex, Hercules, CA, USA) reagent kit. All samples were analyzed using the Luminex X-200, and the data were subsequently analyzed using Milliplex Analyst 5.1. Human Interleukin-37 (IL-37) ELISA Kit (Elabscience, Wuhan, China) was used to detect the expression levels of IL-37 in human serum. All were carried out strictly according to the manufacturer’s instructions, and, after termination of the reaction, the optical density of each well was measured at 450 nm using an enzyme marker.

### 2.9. Statistical Analysis

Continuous variables are expressed as the mean ± standard deviation, while categorical variables are presented as the median (P25-P75). Data following a normal distribution were analyzed using one-way ANOVA, while data not following a normal distribution were analyzed using the chi-square test to compare differences between groups. The relationships between variables were analyzed using Pearson or Spearman correlation analysis, depending on whether the data followed a normal distribution. A multiple linear regression model was used to evaluate the relationships between the variables, including demographic indicators with *p* < 0.1 in inter-group differences (such as gender, duration of local residence, and smoking), as well as COVID-19 and cancer. IL-1β, IL-2, IL-12, IFN-γ, MIP-3α, and TNF-α are defined as pro-inflammatory cytokines, while IL-4, IL-10, IL-13, and IL-37 are defined as anti-inflammatory cytokines. PROCESS (4.1) in SPSS 25.0 was used to analyze the mediation and moderated mediation models, evaluating whether the relationship between urinary fluoride levels and Tregs changes is mediated by cytokines and whether other cytokines regulate the indirect effects in the mediation model. The bias-corrected percentile bootstrap method (Number = 5000) was used to compute the 95% confidence interval (95% CI), with effects considered significant if the confidence interval did not include 0. Data processing and analysis were performed using SPSS 25.0. All hypotheses were tested using a two-tailed test, with *p* < 0.05 considered statistically significant. Figures were generated using GraphPad Prism 8.0.2.

## 3. Results

### 3.1. The Cytokine Network Mediates the Increase of Tregs in the Peripheral Blood of Fluoride-Exposed Populations

#### 3.1.1. Basic Characteristics of the Study Participants and Their Fluoride Accumulation Levels

This study included 327 participants, who were divided into three groups based on urinary fluoride levels: Tertile 1 (low urinary fluoride level, ≤2.08 mg/L), Tertile 2 (moderate urinary fluoride level, >2.08 to ≤3.79 mg/L), and Tertile 3 (high urinary fluoride level, >3.79 mg/L). Table S2 shows that, in the high urinary fluoride level group, there were more male participants, more smokers, a longer residence duration, and a higher cancer incidence compared to the low and moderate urinary fluoride level groups. CD4^+^ and CD8^+^ T-cell counts decreased in a fluoride-dependent manner, suggesting that the immune function of individuals in endemic fluorosis areas may be affected by fluoride exposure.

#### 3.1.2. Long-Term Fluoride Exposure Alters the Immune Microenvironment

**Figure 1:**
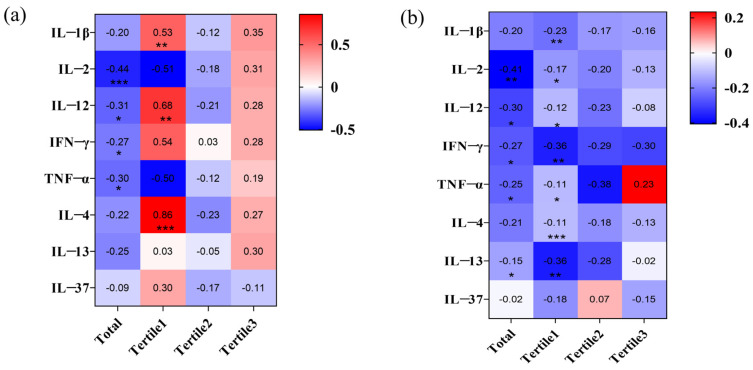
The relationship between urinary fluoride, Tregs, and cytokines: (**a**) The correlation between urinary fluoride and cytokines; and (**b**) the correlation between Tregs and cytokines across low, moderate, and high urinary fluoride levels; *, *p* < 0.05, **, *p* < 0.01, and ***, *p* < 0.001.

Cytokines and Tregs work together to precisely regulate the immune system, participating in the maintenance of immune homeostasis and immune tolerance [39,40,41]. We first measured the cytokine expression levels in the participants to assess the effect of fluoride accumulation on inflammation. Table S3 shows that, in the high urinary fluoride group, the levels of inflammatory cytokines IL-2, IL-12, IFN-γ, and TNF-α, and the anti-inflammatory cytokine IL-13 were significantly lower compared to the low urinary fluoride group. The overall correlation results indicated a negative correlation between urinary fluoride levels and the above cytokines. Interestingly, IL-1β, IL-12, and IFN-γ were moderately to strongly positively correlated with the low urinary fluoride group (Figure 1a). A multiple linear regression analysis of the cytokines with significant group differences (*p* < 0.05) and urinary fluoride levels revealed that, for each one-unit increase in urinary fluoride, the levels of pro-inflammatory cytokines IL-2 (β = −0.303, 95% CI: −0.317, −0.033, *p* = 0.016), IL-12 (β = −0.281, 95% CI: −0.389, −0.031, *p* = 0.022), IFN-γ (β = −0.263, 95% CI: −0.570, −0.031, *p* = 0.029), and TNF-α (β = −0.238, 95% CI: −0.748, −0.008, *p* = 0.046) were significantly reduced (Table 1). In participants from fluoride-poisoned areas, the number of Tregs in the high and moderate urinary fluoride groups was significantly higher than in the low urinary fluoride group (Table S3). A correlation analysis was subsequently conducted between the number of Tregs and urinary fluoride levels, revealing a positive correlation (Figure 1b). The linear regression analysis revealed that, for every 1 mg/L increase in urinary fluoride, the proportion of Tregs in peripheral blood mononuclear cells increased by 0.473% (Table 2). The above results indicate that the accumulation of fluoride ions in the body altered cytokine levels, mediating inflammation and simultaneously recruiting more Tregs into the peripheral circulation to maintain immune homeostasis.

#### 3.1.3. IL-2 and IFN-γ Mediated the Regulation of Tregs by Fluoride

Tregs can reduce inflammation, while the inflammatory environment can affect their quantity and function [42]. How does the network of inflammatory and anti-inflammatory factors affect Tregs after fluoride exposure? Overall, the increase in Tregs is negatively correlated with IL-2, IL-12, IFN-γ, and TNF-α, and also negatively correlated with IL-13, which inhibits inflammation (Figure 1b). When urinary fluoride levels are low (Tertile 1 group), more cytokines are mobilized, affecting the expansion of Tregs, and IL-1β, IL-2, IL-4, IFN-γ, and TNF-α are negatively correlated with Tregs (Figure 1b). After adjusting for confounding factors (Table 3), a multiple linear regression analysis revealed that, for every one-unit increase in urinary fluoride, IL-2 decreased by 0.369% (95% CI: −0.201, −0.043, *p* = 0.003), IL-12 decreased by 0.300% (95% CI: −0.141, −0.016, *p* = 0.014), and IFN-γ decreased by 0.370% (95% CI: −0.100, −0.024, *p* = 0.002).

**Figure 2:**
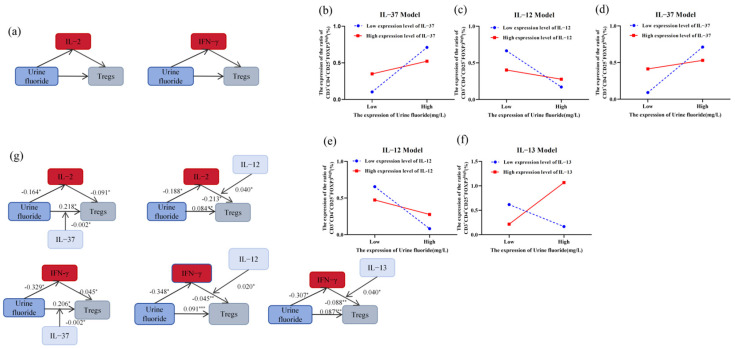
Mediating effects of cytokines, urine fluoride, and Tregs: (**a**) IL-2 and IFN-γ mediate the changes in Tregs quantity induced by fluoride; (**b**) conditional effects of IL-37 as moderator variables on the relationship between fluoride and Tregs through IL-2; (**c**) conditional effects of IL-12 as moderator variables on the relationship between fluoride and Tregs through IL-2; (**d**) conditional effects of IL-37 as moderator variables on the relationship between fluoride and Tregs through IFN-γ; (**e**) conditional effects of IL-12 as moderator variables on the relationship between fluoride and Tregs through IFN-γ; (**f**) conditional effects of IL-13 as moderator variables on the relationship between fluoride and Tregs through IFN-γ; and (**g**) moderated mediation models of IFN-γ or IL-2, fluoride, and Tregs; *, *p* < 0.05, **, *p* < 0.01, and ***, *p* < 0.001.

In summary, IL-2, IL-12, IL-13, IFN-γ, and TNF-α were identified as significantly correlated with urinary fluoride levels and Tregs counts. A mediation analysis further revealed that IFN-γ and IL-2 partially mediated the fluoride-induced elevation in Tregs counts (Table 4, Figure 2a). To further investigate the intricate regulatory mechanisms of cytokines involved in fluoride-induced Tregs recruitment, multiple moderation mediation analyses were conducted on the remaining cytokines to elucidate their potential interactions and roles. The results revealed that IL-12, IL-13, and IL-37 were involved in the indirect effects of fluoride on Tregs (Tables S4–S9). When classified into low (mean − SD), moderate (mean), and high (mean + SD) levels, it was observed that low-to-moderate levels of IL-12, IL-13, and IL-37 significantly modulated the indirect effect of IFN-γ on the fluoride-induced Tregs expansion. Additionally, the low-to-moderate expression of IL-12 and IL-37 influenced the IL-2-mediated indirect effect on the peripheral Tregs expansion induced by fluoride (Table S10, Figure 2b–g).

### 3.2. Effects of Fluoride Exposure on the Immune Microenvironment in Rats

Field investigations have revealed that fluoride influences inflammatory cytokine levels, suggesting the widespread presence of inflammation in the body due to fluoride exposure. Moreover, MIP-3α plays a key role in the migration of Tregs to inflamed tissues [43]. IL-10, as a pleiotropic transcription factor, plays a crucial role in inflammation regulation and is widely involved in the proliferation, development, and functional stability of Tregs [44,45]. Therefore, we included it as a new marker in the rat cytokine detection panel. We selected pro-inflammatory cytokines (IL-1β, IL-2, IL-12, IFN-γ, MIP-3α, and TNF-α) and anti-inflammatory cytokines (IL-4, IL-10, and IL-13) to investigate their effects on the development, differentiation, function, and migration of Tregs in various tissues, as observed in areas with a low-to-moderate fluoride exposure. Additionally, we explored whether reducing the fluoride concentration in drinking water has long-term effects on the immune microenvironment due to the accumulated fluoride in the body.

#### 3.2.1. Fluoride Exposure Has Long-Term Effects on Urinary Fluoride Levels in Rats

**Figure 3:**
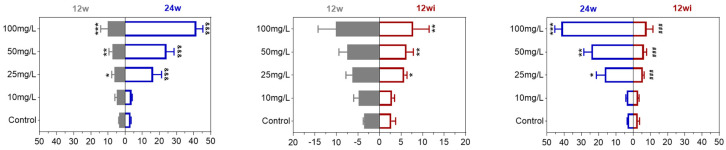
Comparison of urinary fluoride levels at different time points. *, comparison between control; &, comparison of 12-week fluoride treatment group and 24-week fluoride treatment group, and 12-week fluoride treatment and 12-week improvement water treatment group; # comparison of 24-week fluoride treatment group and 12-week fluoride treatment group, and 12-week fluoride treatment and 12-week improvement water treatment group; *, *p* < 0.05, **, *p* < 0.01, ***, *p* < 0.001; ^&&&^, *p* < 0.001; and ^###^, *p* < 0.001.

The results in Figure 3 show that the fluoride concentration in the urine of rats increased with the fluoride dose after 12 and 24 weeks of exposure. Specifically, the urinary fluoride levels in the 25, 50, and 100 mg/L groups were significantly higher than in the control group. Furthermore, after 24 weeks of fluoride exposure, the fluoride levels in the 50 and 100 mg/L groups were significantly higher than after 12 weeks of exposure. Following 12 weeks of being switched to purified water, the urinary fluoride concentrations in the 25 mg/L, 50 mg/L, and 100 mg/L groups remained higher than in the control group. However, no significant difference in urine fluoride was found between the water-improvement group and the 12-week fluoride exposure group, although it was significantly lower than in the 50 and 100 mg/L groups after 24 weeks of fluoride exposure.

#### 3.2.2. Fluoride-Induced Changes in the Proportion of Tregs in Peripheral Blood and Tissues

**Figure 4:**
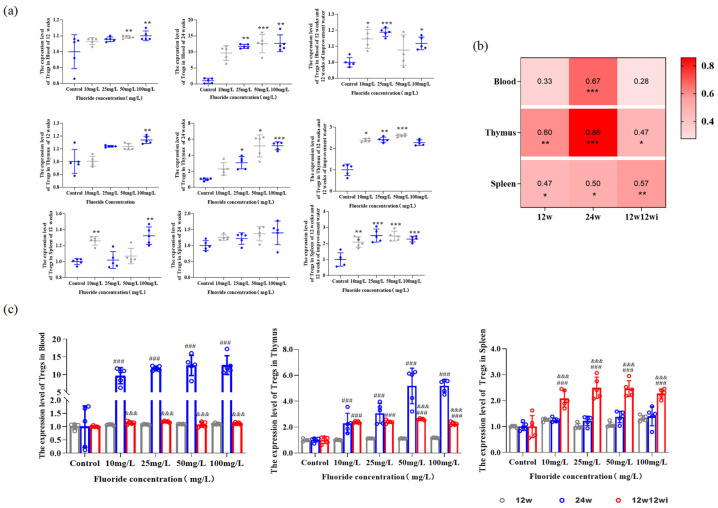
The relationship between fluoride exposure and Tregs changes in peripheral blood and immune organs of rats: (**a**) expression of Tregs in the thymus, blood, and spleen; (**b**) correlation analysis between urinary fluoride and Tregs; (**c**) Tregs expression at different time points and doses—Gray represents 12 w, blue represents 24 w, and red represents 12 w12 wi; *, comparison between control; ^&^, comparison of 12-week fluoride treatment group and 24-week fluoride treatment group, and 12-week fluoride treatment and 12-week improvement water treatment group; ^#^ comparison of 24-week fluoride treatment group and 12-week fluoride treatment group, and 12-week fluoride treatment and 12-week improvement water treatment group; *, *p* < 0.05, **, *p* < 0.01, ***, *p* < 0.001; ^&&&^, *p* < 0.001; and ^###^, *p* < 0.001.

We observed the number of Tregs in the thymus, spleen, and peripheral blood to explore how different fluoride doses and exposure durations affect the immune system homeostasis during inflammation. Figure 4a shows that, after 12 weeks of fluoride exposure, Tregs in the thymus, spleen, and peripheral blood of rats rapidly responded to the inflammatory stimuli in the body induced by fluoride accumulation, with their numbers significantly increasing. Specifically, the number of Tregs in the peripheral blood of the 50 and 100 mg/L groups, in the thymus of the 100 mg/L group, and in the spleen of the 10 and 100 mg/L groups were significantly higher than that in the control group. Interestingly, in the 10 mg/L group after 12 weeks of fluoride exposure, the number of Tregs in the spleen increased and was significantly higher than in the control, 25 mg/L, and 50 mg/L groups. After 24 weeks of fluoride exposure, the number of Tregs in the peripheral blood and thymus of the 25, 50, and 100 mg/L groups significantly increased (Figure 4a). At the 12-week exposure stage, changes in urinary fluoride levels showed a moderate positive correlation with the number of Tregs in immune organs (Figure 4b). After 24 weeks of fluoride exposure, there was a strong correlation between Tregs in the thymus and urinary fluoride levels, and a moderate correlation between the changes in Tregs in the peripheral blood and spleen with urinary fluoride levels (Figure 4b). After 12 weeks of improved water feeding, the number of Tregs in the peripheral blood and immune organs continued to increase with a higher fluoride exposure, with the peripheral blood Tregs significantly higher in the 10, 25, and 100 mg/L groups compared to the control group. The number of Tregs in the spleen increased in the 10, 25, and 50 mg/L groups and showed a moderate positive correlation with urinary fluoride levels. The proportion of thymus-derived Tregs was higher in the fluoride exposure groups than in the control group, with a moderate positive correlation to urinary fluoride levels (Figure 4b).

Figure 4c shows that, after 24 weeks of fluoride exposure, compared to 12 weeks, prolonged fluoride exposure increased the number of Tregs in peripheral blood and the thymus in the 10, 25, 50, and 100 mg/L groups. The time-dependent increase in Tregs indicates that fluoride has a cumulative effect on immune imbalance. After 12 weeks of fluoride exposure followed by 12 weeks of water-improvement, the number of Tregs in the peripheral blood and immune organs of rats was compared with that of the fluoride-only group (Figure 4c). Peripheral blood Tregs were lower in the water-change group compared to the 24-week fluoride exposure group, but no significant difference was observed when compared with the 12-week fluoride exposure group. The number of thymus-derived Tregs was significantly higher than in the 12-week group but lower than in the 24-week group. After 12 weeks of fluoride exposure followed by 12 weeks of improvement water, the number of Tregs in the spleen was significantly higher than in the 12-week and 24-week groups. This suggests that fluoride has a long-term effect on the regulation of Tregs, and, due to fluoride accumulation and slow release in tissues, reducing the fluoride ion concentration in drinking water may cannot mitigate its impact on Tregs numbers.

#### 3.2.3. The Effects of Fluoride Exposure Duration and Dosage on the Expression of Cytokines

**Figure 5:**
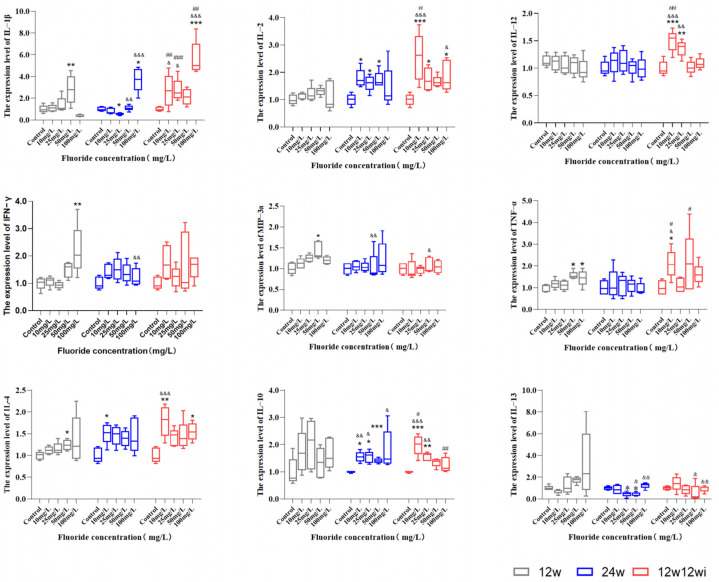
Cytokines expression at different time points and doses. Gray represents 12 w, blue represents 24 w, and red represents 12 w12 wi; *, comparison between control; ^&^, comparison of 12-week fluoride treatment group and 24-week fluoride treatment group, and 12-week fluoride treatment and 12-week improvement water treatment group; ^#^ comparison of 24-week fluoride treatment group and 12-week fluoride treatment group, and 12-week fluoride treatment and 12-week improvement water treatment group; *, *p* < 0.05, **, *p* < 0.01, ***, *p* < 0.001; ^&^, *p* < 0.05, ^&&^, *p* < 0.01, ^&&&^, *p* < 0.001; ^#^, *p* < 0.05, ^##^, *p* < 0.01, ^###^, and *p* < 0.001.

Figure 5 shows that, after 12 weeks of fluoride exposure, the expression levels of pro-inflammatory cytokines IL-1β, IFN-γ, MIP-3α, and TNF-α, and the anti-inflammatory cytokine IL-4 were significantly higher in the high-dose group compared to the control group. Prolonged fluoride exposure exacerbated the inflammatory response in rats. After 24 weeks of fluoride exposure, the expression levels of IL-2 in the middle- and high-dose (25, 50, and 100 mg/L) groups, as well as IL-1β, IFN-γ, and MIP-3α in the high-dose group, were higher than those in the control group. The expression of IL-4 in the 10 mg/L group and IL-10 in the 10, 25, and 50 mg/L groups were significantly higher than in the control group. To further examine the dynamic changes in inflammation induced by fluoride, we compared cytokine levels after different exposure durations. Compared to the 12-week fluoride exposure, prolonging the exposure at the same dose promoted the expression of inflammatory cytokines IL-1β, IL-18, and MIP-3α, and the anti-inflammatory cytokine IL-10, while reducing the levels of IL-13 (Figure 5). The results above show that the levels of IL-4 and IL-10 significantly increased under the 10 mg/L low-dose exposure, indicating that, even at lower fluoride doses, noticeable inflammatory damage can occur. In the 100 mg/L group, only the levels of IL-1β, IFN-γ, TNF-α, IL-10, and IL-13 remained different from those of the control group, while other cytokines showed no significant changes. We hypothesize that this could be due to the progression of inflammatory damage to substantive lesions after prolonged high-dose fluoride exposure.

Figure 5 shows that, after switching to purified water, the expression levels of IL-1β, and IL-2, and the anti-inflammatory factor IL-10 in the 10, 25, and 100 mg/L groups were higher than those in the control group. Additionally, the expression levels of IL-12 in the 10 and 25 mg/L groups, MIP-3α in the 50 mg/L group, TNF-α in the 10 mg/L group, IL-4 in the 10 and 100 mg/L groups, and IL-13 in the 50 and 100 mg/L groups were all higher than those in the control group. Our results show that, after being fed with 10 mg/L fluoride water, even when switched to purified water, the slow release of fluoride ions in the rats still induced changes in the levels of various cytokines (IL-1β, IL-2, IL-12, TNF-α, IL-4, and IL-10). This suggests that even low-dose fluoride exposure has a long-term effect on inflammatory damage in animals.

#### 3.2.4. The Relationship Between Changes in Tregs Numbers in Peripheral Blood and Immune Organs and the Expression of Cytokines

**Figure 6:**
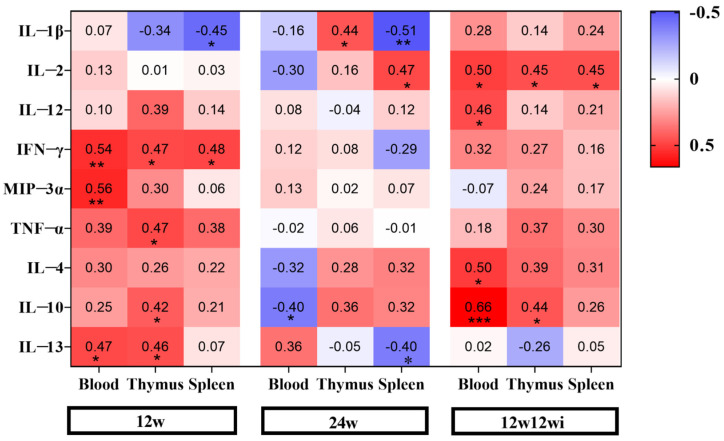
Correlation between cytokines and Tregs. *, *p* < 0.05, **, *p* < 0.01, and ***, *p* < 0.001.

Through a correlation analysis, we explored the potential cytokines that may affect the changes in the number of Tregs in immune organs induced by fluoride. Figure 6 shows that, after 12 weeks of fluoride exposure, IL-1β exhibited a moderate negative correlation with the changes in the number of Tregs in the spleen. IFN-γ showed a moderate correlation with number of Tregs in the peripheral blood, thymus, and spleen. MIP-3α was associated with an increase in peripheral Tregs. TNF-α and IL-10 were correlated with increased thymus Tregs. IL-13 exhibited a positive correlation with a number of Tregs in both the peripheral blood and thymus. After extending the fluoride exposure to 24 weeks, IL-1β was related to the changes in Tregs numbers in both the thymus and spleen, with a positive correlation in the thymus and a negative correlation in the spleen. Spleen Tregs were positively correlated with IL-2 but negatively correlated with IL-13. IL-10 was negatively correlated with peripheral Tregs. After switching to purified water, Tregs in both the peripheral blood and immune organs showed a positive correlation with IL-2. The change in peripheral Tregs numbers was positively correlated with IL-12, IL-4, and IL-10, while the change in thymus-derived Tregs numbers was positively correlated with IL-10. Based on these results, we observed that, after 12 weeks of fluoride exposure, IFN-γ was correlated with changes in Tregs numbers from the peripheral blood and immune organs. After 12 weeks of fluoride exposure, switch to purified water and continue feeding for another 12 weeks; we then found that IL-2 was associated with changes in Tregs numbers in the peripheral blood, thymus, and spleen.

## 4. Discussion

Urine is widely recognized as the primary route for fluoride excretion in both humans and mammals [46,47]. Our findings demonstrate time- and dose-dependent changes in urinary fluoride levels, suggesting that fluoride exposure has a cumulative effect on the body. Moreover, reducing the fluoride concentration in drinking water did not significantly decrease the urinary fluoride levels in rats. This suggests that the long-tail effect of fluoride exposure results in the prolonged exposure of multiple systems and tissues to fluoride. The harmful effects of high-dose fluoride on multiple systems in the body have been well-documented, particularly as various countries have implemented policies to reduce the fluoride concentration in drinking water [48]. The number of high-fluoride exposure regions has gradually decreased, while the number of regions with moderate to low fluoride exposure (<4.0 mg/L) has continued to rise. Therefore, based on the current levels of fluoride exposure and previous studies, we established animal models with fluoride exposures ranging from 10 to 100 mg/L. Based on the dose formula for body surface area between humans and mammals [49], the human equivalent doses for 10, 25, 50, and 100 mg/L were calculated as 1.61, 4.03, 8.06, and 16.12 mg/L, respectively, with 50 and 100 mg/L designated as high-fluoride control groups. The selected doses were aimed at both validating the success of the model and comparing the differences in cytokine-mediated Tregs proportion changes under high- and low-dose fluoride exposure. This dose selection better focuses on the health effects and potential mechanisms of low-dose fluoride exposure (the levels humans encounter in natural environments).

Tregs are key cells in maintaining immune homeostasis. Changes in their proportion and impaired suppressive function regulate the body’s inflammatory response. In turn, this inflammatory environment affects the differentiation and development of Tregs [50]. Although the impact of fluoride on immune homeostasis has been recognized, evidence regarding its regulation of Tregs in inflammatory environments remains limited. Previous studies from our research group have shown that low to moderate fluoride exposure disrupts the immune function in residents of fluoride-exposed areas [22]. Cytokines, small protein molecules produced by immune and non-immune cells in response to stimuli, regulate immune responses and other functions. A systematic description of the characteristic changes in fluoride-induced cytokine alterations, identifying the key cytokines that regulate fluoride-induced immune dysfunction, and explaining the composition of the immune microenvironment and the critical role of cytokines in Tregs regulation, may provide valuable insights into improving the immune function in residents from areas with low to moderate fluoride exposure.

A cross-sectional study from Pakistan reported that fluoride is a risk factor for inflammation in areas with prevalent fluoride exposure [51]. Extensive evidence from field surveys and animal models has consistently confirmed a significant positive correlation between high fluoride exposure and inflammation [52,53,54]. To explore which cytokines are involved in the regulation of Tregs by fluoride in an inflammatory environment, we systematically analyzed the expression of cytokines in the serum of adults from areas with low-to-moderate fluoride exposure (0.89–2.66 mg/L). We found that, even at low levels of fluoride accumulation in the body, inflammatory responses are induced, providing evidence that low-dose fluoride also induces inflammation in the body. In individuals with high urinary fluoride levels, the serum levels of IL-2, IL-12, IFN-γ, and TNF-α were found to be lower. This may be because low fluoride levels promote inflammation, and, as fluoride accumulates in the body, the tissues progress from an inflammatory state to functional impairment. This consumes more cytokines involved in immune cell recruitment and function maintenance. Additionally, cytokine-secreting cells may undergo apoptosis or functional damage due to inflammatory injury, leading to a decrease in cytokine expression. Epidemiological surveys and animal experiments have also reported that high doses of fluoride induce a decrease in the expression levels of cytokines such as IL-2, IFN-γ, and TNF-α in serum [55,56] In recent years, it has been widely reported that the IL family, IFN-γ, TNF-α, and other cytokines are involved in fluoride-induced tissue inflammation in mammals [20,57,58,59]. However, the dynamic changes of these cytokines in relation to fluoride exposure time and dose, as well as the potential long-term effects, remain unknown. This animal study systematically demonstrates that prolonged fluoride exposure recruits a greater variety and number of inflammatory factors into the rat’s inflammatory environment, while also activating more anti-inflammatory factors to counteract the damage. The results of animal experiments after water improvement showed that, even after the fluoride concentration in drinking water was reduced, they still excreted fluoride with urine—so ingested fluoride was still influencing the immune system. Therefore, we believe that the immunotoxicity of fluoride has long-term effects.

An increasing body of evidence suggests that the inflammatory environment is a key determinant of immunotherapy efficacy, and Tregs reprogramming in inflammation significantly affects the disease course and prognosis [60]. Our field investigation and animal experiments show that fluoride exposure promotes the recruitment of Tregs in immune organs, suggesting that fluoride accumulation intensifies the inflammatory response, prompting the immune system to recruit more Tregs into the peripheral circulation to maintain the immune homeostasis. However, after 12 weeks of drinking fluoride-containing water, the Tregs count in the spleen of the 10 mg/L group was higher than that of the 25 and 50 mg/L groups. This result further confirms our previous finding that higher doses of fluoride (25 and 50 mg/L) inhibit the proliferation of T lymphocytes in the spleen and promote the apoptosis of spleen lymphocytes [28]. During inflammation, tissue-resident Tregs are depleted to exert their suppressive functions and promote tissue repair. Additionally, our study is the first to observe in an animal model that, even after fluoride exposure is reduced and the animals are switched to purified water, Tregs continue to accumulate in the peripheral blood and immune organs. This indicates that the rats’ immune systems are not capable of fully restoring immune homeostasis on their own. Therefore, identifying the mediators of the fluoride-induced immune imbalance and understanding the relationship between inflammation and dysregulated Tregs will help identify effective key factors for restoring the immune homeostasis and enhancing immune function. Both our field investigation and animal experiments have found that cytokines are involved in the changes in Tregs proportions induced by fluoride exposure. The development and functional stability of Tregs rely on cytokines [41], among which the IL family, IFN-γ, and TNF-α are essential factors for driving Tregs activation and the expression of their suppressive functions [61,62,63].

It is well-known that Tregs require IL-2 to maintain their numbers, development, and immune function [64]. Currently, considerable attention has been directed towards the regulation of Tregs by low-dose IL-2 (<1.5 million IU) [65]. Low-dose IL-2 is an effective way to increase the number and function of Tregs in peripheral circulation [66,67]. This study found that IL-2 partially mediates the fluoride-induced changes in the Tregs quantity in the body’s internal environment. Additionally, the increase in Tregs was associated with a low IL-2 expression in the subjects. After switching to purified water, the elevation of Tregs in both the peripheral blood and immune organs was linked to IL-2, suggesting that IL-2 may play an unexpected role in the regulation of Tregs by fluoride. This suggests that, although the long-term survival of Tregs depends on continuous IL-2 signaling [68], in animal experiments, the number of Tregs in the peripheral blood and immune organs did not exhibit IL-2-dependent changes. However, this does not imply that IL-2’s role in rat Tregs is negligible. This may be because continuous IL-2 signaling is primarily involved in maintaining the suppressive function of rat Tregs rather than their proliferation [68]. Based on this result, we hypothesize that, under the influence of fluoride, the Tregs accumulated in the peripheral blood and immune organs may be dysfunctional.

We also found that other members of the IL family (IL-4, IL-13, and IL-10) are associated with changes in Tregs numbers. Some studies suggest that T cells exposed to both IL-2 and IL-4 environments promote Tregs proliferation more effectively than exposure to either cytokine alone [69]. In our post-water change animal model, we also found that changes in the number of peripheral-blood-derived Tregs were correlated with both IL-2 and IL-4. This suggests that the abnormal Tregs number induced by fluoride exposure may be the result of a combined regulation by multiple cytokines. Additionally, higher levels of IL-13 recruit more Tregs compared to lower levels [70]. IL-10, as an anti-inflammatory cytokine, plays a crucial role in Tregs’ suppressive function and the maintenance of immune tolerance [71,72]. Our results show that IL-10 is widely involved in the changes in Tregs numbers from peripheral blood and immune organs. This further suggests that fluoride not only recruits Tregs in terms of quantity but also exerts functional suppression on them.

IFN-γ is considered to play a crucial role in driving inflammation and regulating immune homeostasis, inducing self-regulatory mechanisms in the inflammatory microenvironment [73]. Our results indicate that IFN-γ is associated with changes in Tregs numbers in both the fluoride-exposed population and rats. Animal results show that IFN-γ is associated with the increase in Tregs in both peripheral blood and immune organs. As a pleiotropic cytokine, IFN-γ exhibits dual effects on Tregs activity: some studies report that IFN-γ inhibits the activation and proliferation of Tregs in humans and mice [74], while other research suggests that the absence of IFN-γ weakens the expression of Foxp3, a key factor regulating Tregs’ suppressive function, thereby limiting their suppressive capacity [75]. Our results suggest that, in the inflammatory environment affected by fluoride, a decrease in IFN-γ is associated with an increase in the proportion of peripheral Tregs. In addition, a recent study suggests that IFN-γ, in combination with IL-12, is involved in the activation and proliferation of Tregs [76]. IL-12 is an indispensable key factor for the activation of Tregs and also plays a role in stabilizing their regulatory function during the activation process [77]. Our field investigation also found that IL-12 is involved in the indirect effect of IFN-γ on fluoride-induced changes in the proportion of Tregs. The combination of IFN-γ and IL-12 may contribute to the impact of fluoride on Tregs numbers and functional homeostasis. Our field investigation also found that IL-12 is involved in the indirect effect of IFN-γ on fluoride-induced changes in the proportion of Tregs. The combination of IFN-γ and IL-12 may contribute to the impact of fluoride on Tregs numbers and functional homeostasis.

Studies have reported that the production of chemokines is crucial for the migration of Tregs [78]. The accumulation of Tregs in both lymphoid and non-lymphoid tissues is influenced by the expression of chemokines, which participate in the process of Tregs migrating from the periphery to non-lymphoid organs [79]. Among these chemokines, MIP-3α plays a role in the migration of Tregs to non-immune organs such as the intestine, brain, and liver [80,81,82,83]. Our study first revealed that fluoride accumulation affects MIP-3α levels and is associated with changes in Tregs numbers in the peripheral circulation. This suggests that, under the influence of MIP-3α, fluoride may regulate the migration of Tregs to non-immune organs.

TNF-α has a broad and profound impact on the activation and proliferation of different immune cell subsets. Studies have shown that blocking TNF-α in vitro can promote Tregs proliferation [84]. In mice, TNF-α positively regulates Tregs function [85], as evidenced by our study, where, after 12 weeks of improved water treatment, the serum TNF-α levels in rats increased, indicating the initiation of Tregs function repair. However, the regulatory effect of TNF-α on human Tregs remains controversial, which may be attributed to its two receptors: the pro-inflammatory TNFR-1 and the anti-inflammatory TNFR-2 [86]. Therefore, the proliferation of Tregs in the peripheral blood of participants and the decline in TNF-α levels may be due to the opposing effects of TNF-α at different stages of fluoride-induced inflammation.

This study found that IL-2 and IFN-γ are associated with changes in Tregs numbers regulated by fluoride in the body environment. Through a rat model, we further investigated the correlation between cytokines and Tregs in various organs, and found that different cytokines may dynamically regulate Tregs changes at different stages of fluoride exposure. However, the mechanisms by which cytokines exert their effects are complex, involving intricate signaling pathways and regulatory networks. Further exploration is needed to understand the complex interactions between cytokines and the molecular mechanisms underlying changes in Tregs numbers. This will be a focus of our future research. Additionally, in this study, the 12-week fluoride exposure group, the 24-week fluoride exposure group, and the 12-week fluoride exposure followed by 12-week improved water treatment group were designed to better simulate real-world fluoride exposure in endemic fluorosis areas. However, the biological differences and genetic diversity between animals and humans in this study may lead to discrepancies between the study results and their actual application. With the reduction in fluoride levels in drinking water, understanding the relationship between fluoride-regulated cytokines and immune function and using intervention strategies to maintain immune function and promote inflammation repair become particularly important. Our findings not only provide new immune checkpoints for the early monitoring of endemic fluorosis but also broaden the approach for maintaining immune function and promoting inflammation repair after fluoride toxicity.

## 5. Conclusions

Our study demonstrates that even low-dose fluoride can induce the disruption of the inflammatory microenvironment in both humans and rats. Fluoride accumulation in the bodies of residents in endemic fluorosis areas and the recruitment of Tregs in the periphery may be regulated by cytokines, a regulatory mechanism that was also validated in the fluorosis rat model. This finding suggests that the combination therapy of cytokines and Tregs could play a crucial role during the inflammatory phase of fluorosis. These cytokines, which modulate fluoride-induced changes in Tregs in the inflammatory environment, may represent untapped new avenues for immune therapy. Moreover, reducing the fluoride concentration in drinking water alone cannot fully restore the immune imbalance and inflammation damage in rats, indicating that the effects of fluoride on the body have both cumulative and long-term consequences. This suggests that, in subsequent on-site investigations and laboratory studies, we should not only focus on the health impacts of long-term high fluoride exposure, but also prioritize research on the long-term health impacts after reducing the fluoride concentration in drinking water following prolonged fluoride exposure.

## Figures and Tables

**Table 1:** Results of multiple linear regression for the relationship between urinary fluoride and cytokines.

UF (mg/L)	Crude, β (95% CI) ^a^	*p*	Adjusted, β (95% CI) ^a^	*p*
	IL-2			
Tertile1 (≤2.08 mg/L) ^b^	0.241 (−1.230, 3.394)	0.336	0.284 (−1.370, 3.926)	0.318
Tertile2 (>2.08–≤3.79 mg/L) ^c^	−0.101 (0.654, −1.503)	0.654	−0.155 (−1.732, 0.905)	0.517
Tertile3 (>3.79 mg/L) ^b^	0.230 (−0.100, 0.303)	0.303	0.302 (−0.102, 0.370)	0.247
Total ^c^	−0.303 (−0.317, −0.033)	0.016	−0.294 (−0.314, −0.026)	0.022
	IL-12			
Tertile1 (≤2.08 mg/L) ^b^	0.299 (−0.830, 4.544)	0.165	1.367 (−0.981, 4.741)	0.185
Tertile2 (>2.08–≤3.79 mg/L) ^c^	−0.194 (−2.069, 0.837)	0.387	−0.24 (−2.413, 0.887)	0.343
Tertile3 (>3.79 mg/L) ^b^	0.243 (−0.109, 0.346)	0.289	0.37 (−0.082, 0.443)	0.165
Total ^c^	−0.281 (−0.389, −0.031)	0.022	−0.291 (−0.402, −0.033)	0.022
	IFN-γ			
Tertile1 (≤2.08 mg/L) ^b^	0.140 (−2.772, 5.271)	0.525	0.153 (−2.858, 5.597)	0.506
Tertile2 (>2.08–≤3.79 mg/L) ^c^	0.104 (−1.862, 2.979)	0.636	0.049 (−2.396, 2.919)	0.838
Tertile3 (>3.79 mg/L) ^b^	0.277 (−0.135, 0.608)	0.200	0.352 (−0.139, 0.739	0.169
Total ^c^	−0.263 (−0.570, −0.031)	0.029	−0.268 (−0.578, −0.035)	0.027
	TNF-α			
Tertile1 (≤2.08 mg/L) ^b^	0.045 (−4.817, 5.932)	0.832	0.028 (−5.251, 5.949)	0.898
Tertile2 (>2.08–≤3.79 mg/L) ^c^	−0.088 (−3.715, 2.500)	0.688	−0.069 (−3.968, 3.024)	0.780
Tertile3 (>3.79 mg/L) ^b^	0.240 (−0.292, 0.989)	0.270	0.181 (−0.493, 1.020)	0.474
Total ^c^	−0.238 (−0.748, −0.008)	0.046	−0.248 (−0.772, −0.016)	0.041
	IL-13			
Tertile1 (≤2.08 mg/L) ^b^	0.117 (−0.673, 1.179)	0.577	0.145 (−0.636, 1.264)	0.500
Tertile2 (>2.08–≤3.79 mg/L) ^c^	−0.025 (−9.130, 8.189)	0.911	0.127 (−6.724, 11.580)	0.584
Tertile3 (>3.79 mg/L) ^b^	0.381 (−0.007, 0.147)	0.073	0.351 (−0.028, 0.157)	0.161
Total ^c^	−0.042 (−0.608, 0.429)	0.710	−0.052 (−0.633, 0.409)	0.668

β, Standardization regression coefficient; CI, confidence interval; *p*: *p*-value; UF, urinary fluoride. ^a^ The assessments of β and 95% CI for every 1 mg/L increment of urine fluoride. ^b^ Adjustment: hypertension. ^c^ Adjustment: Smoke, hypertension, cancer.

**Table 2:** Results of correlation and logistic regression analysis for the relationship between urinary fluoride and Tregs.

UF (mg/L)	Tregs					
	Crude, β (95% CI) a	*p* ^d^	Adjusted, β (95% CI) ^a^	*p* ^d^	R	*p* ^e^
Tertile1 (≤2.08 mg/L) ^b^	−0.040 (−0.111, 0.093)	0.857	0.002 (−0.283, 0.285)	0.994	0.248	0.232
Tertile2 (>2.08–≤3.79 mg/L) ^c^	−0.166 (−0.436, 0.200)	0.448	−0.311 (−0.600, 0.158)	0.233	−0.156	0.477
Tertile3 (>3.79 mg/L) ^b^	−0.030 (−0.235, 0.204)	0.887	0.012 (−0.126, 0.150)	0.855	0.091	0.681
Total ^c^	0.547 (0.066, 0.143)	<0.001	0.473 (0.048, 0.133)	<0.001	0.547	<0.001

β, Standardization regression coefficient; CI, confidence interval; *p*: *p*-value; R, correlation coefficient; UF, urine fluoride. ^a^ The assessments of β and 95% CI for every 1 mg/L increment of urine fluoride. ^b^ Adjustment: Gender, duration of local residence, smoke, COVID-19, hypertension. ^c^ Adjustment: Gender, duration of local residence, smoke, COVID-19, hypertension, cancer. ^d^ *p*-value of linear regression results. ^e^ *p*-value of correlation between Tregs and urinary fluoride.

**Table 3:** Results of multiple linear regression for conversion of cytokines and Tregs.

UF (mg/L)	Crude, β (95% CI) ^a^	*p*	Adjusted, β (95% CI) ^a^	*p*
	IL-2			
Tertile1 (≤2.08 mg/L) ^b^	−0.082 (−0.078, 0.057)	0.745	−0.066 (−0.078, 0.061)	0.798
Tertile2 (>2.08–≤3.79 mg/L) ^c^	−0.244 (−0.186, 0.056)	0.274	−0.338 (−0.226, 0.045)	0.178
Tertile3 (>3.79 mg/L) ^b^	−0.166 (−0.322, 0.152)	0.461	−0.168 (−0.341, 0.169)	0.486
Total ^c^	−0.369 (−0.201, −0.043)	0.003	−0.384 (−0.209, −0.045)	0.003
	IL-12			
Tertile1 (≤2.08 mg/L) ^b^	0.35 (−0.005, 0.051)	0.101	−0.091 (−0.246, 0.159)	0.657
Tertile2 (>2.08–≤3.79 mg/L) ^c^	−0.251 (−0.157, 0.045)	0.261	−0.318 (−0.179, 0.037)	0.183
Tertile3 (>3.79 mg/L) ^b^	−0.128 (−0.269, 0.155)	0.581	0.124 (−0.449, 0.716)	0.635
Total ^c^	−0.3 (−0.141, −0.016)	0.014	−0.315 (−0.145, −0.020)	0.011
	IFN-γ			
Tertile1 (≤2.08 mg/L) ^b^	0.165 (−0.016, 0.036)	0.450	−0.043 (−0.309, 0.257)	0.849
Tertile2 (>2.08–≤3.79 mg/L) ^c^	−0.368 (−0.105, 0.007)	0.084	−0.137 (−0.427, 0.215)	0.495
Tertile3 (>3.79 mg/L) ^b^	−0.266 (−0.186, 0.045)	0.219	−0.041 (−0.543, 0.458)	0.871
Total ^c^	−0.370 (−0.100, −0.024)	0.002	−0.394 (−0.105, −0.027)	0.001
	TNF-α			
Tertile1 (≤2.08 mg/L) ^b^	0.268 (−0.006, 0.028)	0.194	0.242 (−0.008, 0.028)	0.264
Tertile2 (>2.08–≤3.79 mg/L) ^c^	0.021 (−0.084, 0.002)	0.059	−0.457 (−0.093, −0.002)	0.043
Tertile3 (>3.79 mg/L) ^b^	0.234 (−0.032, 0.104)	0.283	0.238 (−0.038, 0.112)	0.317
Total ^c^	−0.202 (−0.053, 0.004)	0.091	−0.214 (−0.055, 0.003)	0.080
	IL-13			
Tertile1 (≤2.08 mg/L) ^b^	0.306 (−0.025, 0.169)	0.137	0.379 (−0.010, 0.189)	0.076
Tertile2 (>2.08–≤3.79 mg/L) ^c^	−0.092 (−0.020, 0.013)	0.675	−0.053 (−0.021, 0.017)	0.834
Tertile3 (>3.79 mg/L) ^b^	0.074 (−0.463, 0.645)	0.737	0.066 (−0.522, 0.684)	0.782
Total ^c^	−0.064 (−0.027, 0.016)	0.597	−0.087 (−0.030, 0.014)	0.487

β, standardization regression coefficient; CI, confidence interval; *p*: *p*-value; UF, urinary fluoride. ^a^ The assessments of β and 95% CI for every 1 mg/L increment of urine fluoride. ^b^ Adjustment: hypertension. ^c^ Adjustment: Smoke, hypertension, cancer.

**Table 4:** The mesomeric effect of IFN-γ or IL-2 on the relationship of urine fluoride and Tregs.

Variables	Path a			Path b and c′			Path c			Path a ∗ b			
	β	SE	*p*	β	SE	*p*	β	SE	*p*	β	Boot SE	Boot LLCI	Boot ULCI
Urinary fluoride	−0.294	0.072	0.022	0.423	0.022	<0.001	0.499	0.022	<0.001	0.076	0.039	0.010	0.162
IL-2				−0.257	0.039	0.034							
R2	0.134			0.323			0.266						
F	2.207			5.338			5.158						
Urinary fluoride	−0.280	0.137	0.022	0.452	0.02	<0.001	0.528	0.020	<0.001	0.076	0.034	0.017	0.148
IFN-γ				−0.27	0.018	0.013							
R2	0.097			0.359			0.294						
F	2.330			8.973			9.002						

β, Standardization regression coefficient; *p*: *p*-value. ∗, Multiplication sign.

## Data Availability

The original contributions presented in this study are included in this article. Further inquiries can be directed to the corresponding author.

## Supplementary Materials

The following supporting information can be downloaded at: https://www.mdpi.com/article/10.3390/toxics13020095/s1, Table S1: Building animal models; Table S2: Baseline characteristics across stages of Urinary fluoride; Table S3: Comparison of intergroup differences in different cytokines; Table S4: Moderated mediation analysis-Model 5 (IFN-γ); Table S5: Moderated mediation analysis of other cytokines-Model 5 (IL-2); Table S6: Moderated mediation analysis of other cytokines-Model 7 (IFN-γ); Table S7: Moderated mediation analysis of other cytokines-Model 7(IL-2). Table S8: Moderated mediation analysis of other cytokines-Model 14(IFN-γ); Table S9: Moderated mediation analysis of other cytokines-Model 14(IL-2); and Table S10: Conditional direct effects of urinary fluoride on Tregs at values of IFN-γ, and IL-2.
