# Supplementary material for: Fluoride-Mediated Immune Damage Through Cytokine Network Regulation of Tregs
Source: Toxics. 2025 Jan 26;13(2):95. doi: 10.3390/toxics13020095 (PMC11861542; doi:10.3390/toxics13020095)
Supplement: Supplementary file 1 [file toxics-13-00095-s001.zip › toxics-3430225-supplementary.pdf]

Supplemental Material:

Table S1.

Building animal models

Wistar Rats N=30		
Raising for 12 weeks And then euthanize *n*=10 (12 w)	Continue to raise for 12 weeks and then euthanize N=20	
	The original experimental conditions were continued *n*=10 (24 w)	Change to pure water breeding *n*=10 (12w12wi)
Control	Control	Control
10mg/L(Low-dose)	10mg/L	10mg/L+Improvement water
25mg/L(Middle-dose)	25mg/L	25mg/L+Improvement water
50mg/L(Middle-dose)	50mg/L	50mg/L+Improvement water
100mg/L(High-dose)	100mg/L	100mg/L+Improvement water

Table S2.

Baseline characteristics across stages of Urinary fluoride.

Variable	Urinary Fluoride (mg/L)			P [d]
	Tertile 1 ≤2.08	Tertile 2 >2.08-≤3.79	Tertile 3 >3.79	
Sample Size	109	109	109	
Age[c] (years)	61 (49.5-68)	61 (55-66.5)	61 (55-68)	0.365
Alcohol[a]				0.898
Yes	13 (11.9%)	15 (13.8%)	15 (13.8%)	
No	96 (88.1%)	94 (86.2%)	94 (86.2%)	
Allergy[a]				0.119
Yes	5 (4.6%)	12 (11.0%)	9 (8.2%)	
No	100 (91.7%)	87 (79.8%)	89 (81.7%)	
Unclear	4 (3.7%)	10 (9.2%)	11 (10.1%)	
Anodyne[a]				0.208
Never	96 (88.1%)	89 (81.7%)	91 (83.5%)	
Occasionally	10 (9.2%)	14 (12.8%)	17 (15.6%)	
Often	3 (2.7%)	6 (5.5%)	1 (0.9%)	
BMI[c] (kg/m^2)	25.90 (23.55-28.60)	25.20 (23.15-28.10)	25.40 (22.20-27.20)	0.137
Cancer[a]				**<0.050**
Yes	0	2 (1.9%)	5 (4.6%)	
No	109 (100%)	107 (98.1%)	104 (95.4%)	
$CD4^+$	30.22±5.38	19.85±6.82	9.72±2.52	**<0.001**
$CD8^+$	11.27±3.62	5.27±3.08	0.54±0.53	**<0.001**
COVID-19 [a]				0.068
Yes	65 (59.6%)	58 (53.2%)	46 (42.2%)	
No	42 (38.5%)	46 (42.2%)	55 (50.5%)	
Unclear	2 (1.9%）	5 (4.6%)	8 (7.3%)	
Duration of local residence [b] (years)	49.30±18.33	55.23±13.53	57.99±12.46	**<0.001**
Gender[a]				**<0.001**
Male	27 (24.8%)	41 (37.6%)	51 (46.8%)	
Female	82 (75.2%)	68 (62.4%)	58 (53.2%)	

Hypertension[a]				0.087
Yes	30 (27.5%)	34 (31.2%)	19（17.4%）	
No	79 (72.5%)	75 (68.8%)	90 (83.6%)	
Water[a]				0.281
Tap water	30 (27.5%)	26 (23.9%)	27 (24.8%)	
Well water	65 (59.6%)	77 (70.6%)	76 (69.7%)	
Cellaring water	1 (0.9%)	0	0	
Others	13 (11.9%)	6 (5.5%)	6 (5.5%)	
Water intake [c] (mL)	1000 (650-1500)	1000 (555-1500)	1000 (500-1500)	0.473
Smoke[a]				**<0.050**
Yes	14 (12.8%)	22 (20.2%)	26 (23.9%)	
No	95 (87.2%)	87 (79.8%)	83 (76.1%)	

P: *P*-value.

[a] Number (percentage) for categorical variables.

[b] Data were presented as mean±standard deviation for continuous variables.

[c] Data were presented as P25-P75 for continuous variables.

[d] Student's t-test or Mann-Whitney U test was used to compare the difference of continuous variables, and Chi-square test or Chi-square test for trend was applied to compare the difference of categorical variable

Table S3.

Comparison of intergroup differences in different cytokines

Variables	Urinary Fluoride (mg/L)			P [c]
	Tertile 1 ≤2.08	Tertile 2 >2.08-≤3.79	Tertile 3 >3.79	
IL-1β	0.226±0.160	0.168±0.092	0.149±0.082	0.140
IL-2	2.468±1.638	2.041±1.247	1.497±0.897*	0.047
IL-12	4.156±6.089	3.830±7.274	1.513±1.260*	0.070
IFN-γ	4.638±3.151	3.768±2.466	2.367±1.765*	0.019
TNF-α	8.768±4.182	7.432±3.162	5.623±2.971***	0.019
IL-4	0.610±0.472	0.501±0.334	0.343±0.317	0.083
IL-13	0.755±0.725	0.433±0.2529	0.405±0.376*	0.046
IL-37	73.418±26.030	73.120±35.870	68.767±30.702	0.844
Tregs	0.124±0.171	0.366±0.327***	0.827±0.463***	<0.001

P: *P*-value.

[a] Data were presented as mean±standard deviation for continuous variables.

[b]·Compared with the Tertile 1 group, $^{*}P<0.05$, $^{***}P<0.001$

[c] Student's t-test or Mann-Whitney U test was used to compare the difference of continuous varia

Table S4.

Moderated mediation analysis ——Model 5 (IFN-γ)

Outcome	Predictor	B	SE	*P*	LLCI	ULCI
Tregs	Urinary fluoride	0.088	0.043	0.048	0.001	0.174
	IFN-γ	-0.083	0.029	0.005	-0.140	-0.025
	IL-1β	1.015	0.839	0.231	-0.663	2.694
	Urinary fluoride*IL-1β	-0.006	0.24	0.981	-0.486	0.474
IFN-γ	Urinary fluoride	-0.307	0.136	0.027	-0.578	-0.035
Tregs	Urinary fluoride	0.111	0.047	0.023	0.016	0.206
	IFN-γ	-0.063	0.034	0.065	-0.130	0.004
	IL-2	0.068	0.085	0.428	-0.103	0.238
	Urinary fluoride*IL-2	-0.019	0.021	0.363	-0.061	0.023
IFN-γ	Urinary fluoride	-0.388	0.138	0.007	-0.665	-0.111
Tregs	Urinary fluoride	0.130	0.040	0.002	0.049	0.211
	IFN-γ	-0.058	0.031	0.069	-0.120	0.005
	IL-12	0.069	0.061	0.270	-0.055	0.192
	Urinary fluoride*IL-12	-0.020	0.017	0.246	-0.055	0.014
IFN-γ	Urinary fluoride	-0.348	0.141	0.016	-0.629	-0.066
Tregs	Urinary fluoride	0.103	0.046	0.029	0.011	0.195
	IFN-γ	-0.074	0.028	0.011	-0.130	-0.018
	TNF-α	0.032	0.029	0.266	-0.025	0.090
	Urinary fluoride*TNF-α	-0.002	0.006	0.770	-0.013	0.010
IFN-γ	Urinary fluoride	-0.307	0.136	0.027	-0.578	-0.035
Tregs	Urinary fluoride	0.124	0.036	0.001	0.051	0.196
	IFN-γ	-0.051	0.034	0.134	-0.119	0.016
	IL-4	0.270	0.285	0.346	-0.299	0.839
	Urinary fluoride*IL-4	-0.074	0.060	0.220	-0.194	0.046
IFN-γ	Urinary fluoride	-0.307	0.136	0.027	-0.578	-0.035
Tregs	Urinary fluoride	0.096	0.031	0.003	0.033	0.159
	IFN-γ	-0.045	0.019	0.017	-0.082	-0.008

	IL-13	0.039	0.112	0.729	-0.185	0.262
	Urinary fluoride*IL-13	-0.015	0.039	0.701	-0.093	0.063
IFN-γ	Urinary fluoride	-0.307	0.136	0.027	-0.578	-0.035
Tregs	Urinary fluoride	**0.206**	**0.059**	**0.001**	**0.087**	**0.324**
	IFN-γ	**-0.045**	**0.018**	**0.012**	**-0.08**	**-0.01**
	IL-37	**0.006**	**0.003**	**0.049**	**0.000**	**0.012**
	Urinary fluoride*IL-37	**-0.002**	**0.001**	**0.044**	**-0.003**	**-0.001**
IFN-γ	Urinary fluoride	**-0.329**	**0.146**	**0.028**	**-0.621**	**-0.036**

B, regression coefficient; SE, standard deviation; *P*: *P*-value; LLCI、ULCI, confidence interval

[a] Adjustment: Smoke,hypertension,Cancer.

Table S5.

Moderated mediation analysis of other cytokines——Model 5 (IL-2).

Outcome	Predictor	B	SE	*P*	LLCI	ULCI
Tregs	Urinary fluoride	0.070	0.048	0.148	-0.026	0.165
	IL-2	-0.148	0.068	0.032	-0.284	-0.013
	IL-1β	0.633	0.943	0.505	-1.258	2.524
	Urinary fluoride*IL-1β	0.072	0.258	0.781	-0.446	0.590
IL-2	Urinary fluoride	-0.170	0.072	0.022	-0.314	-0.026
Tregs	Urinary fluoride	0.125	0.045	0.008	0.034	0.217
	IL-2	-0.083	0.087	0.342	-0.258	0.091
	IL-12	0.052	0.086	0.553	-0.122	0.225
	Urinary fluoride*IL-12	-0.020	0.019	0.294	-0.058	0.018
IL-2	Urinary fluoride	-0.188	0.075	0.015	-0.338	-0.039
Tregs	Urinary fluoride	0.128	0.045	0.006	0.039	0.217
	IL-2	0.017	0.063	0.790	-0.110	0.144
	IFN-γ	-0.020	0.046	0.667	-0.112	0.073
	Urinary fluoride* IFN-γ	-0.016	0.011	0.16	-0.038	0.006
IL-2	Urinary fluoride	-0.170	0.072	0.022	-0.314	-0.026
Tregs	Urinary fluoride	0.076	0.054	0.163	-0.032	0.184
	IL-2	-0.120	0.069	0.087	-0.257	0.018
	TNF-α	0.013	0.037	0.728	-0.060	0.086
	Urinary fluoride*TNF-α	0.001	0.007	0.864	-0.012	0.014
IL-2	Urinary fluoride	-0.170	0.072	0.022	-0.314	-0.026
Tregs	Urinary fluoride	0.102	0.042	0.020	0.017	0.187
	IL-2	-0.004	0.106	0.969	-0.217	0.209
	IL-4	-0.179	0.474	0.708	-1.128	0.771
	Urinary fluoride*IL-4	-0.042	0.068	0.543	-0.179	0.095
IL-2	Urinary fluoride	-0.170	0.072	0.022	-0.314	-0.026
Tregs	Urinary fluoride	0.08	0.035	0.026	0.010	0.150
	IL-2	-0.085	0.040	0.038	-0.164	-0.005

	IL-13	-0.007	0.119	0.953	-0.247	0.232
	Urinary fluoride*IL-13	0.001	0.042	0.985	-0.082	0.084
IL-2	Urinary fluoride	-0.170	0.072	0.022	-0.314	-0.026
Tregs	Urinary fluoride	0.218	0.069	0.003	0.081	0.356
	IL-2	-0.091	0.039	0.024	-0.171	-0.012
	IL-37	0.008	0.004	0.036	0.001	0.015
	Urinary fluoride*IL-37	-0.002	0.001	0.044	-0.004	0
IL-2	Urinary fluoride	-0.164	0.078	0.041	-0.320	-0.007

B, regression coefficient; SE, standard deviation; *P*: *P*-value; LLCI、ULCI, confidence interval

Adjustment: Smoke, hypertension, Cancer.

Table S6.

Moderated mediation analysis of other cytokines——Model 7 (IFN-γ).

Outcome	Predictor	B	SE	*P*	LLCI	ULCI
IFN-γ	Urinary fluoride	-0.155	0.191	-0.808	0.422	-0.537
	IL-1β	15.69	3.148	4.985	0.000	9.398
	Urinary fluoride*IL-1β	0.175	1.065	0.164	0.87	-1.954
Tregs	Urinary fluoride	0.087	0.021	4.238	0.000	0.046
	IFN-γ	-0.045	0.018	-2.490	0.015	-0.082
IFN-γ	Urinary fluoride	-0.238	0.187	0.209	-0.614	0.137
	IL-2	1.374	0.288	<0.001	0.797	1.951
	Urinary fluoride*IL-2	0.057	0.084	0.503	-0.112	0.225
Tregs	Urinary fluoride	0.072	0.022	0.002	0.028	0.116
	IFN-γ	-0.059	0.020	0.004	-0.098	-0.020
IFN-γ	Urinary fluoride	-0.172	0.171	0.318	-0.514	0.170
	IL-12	1.031	0.224	<0.001	0.582	1.480
	Urinary fluoride*IL-12	0.067	0.074	0.369	-0.081	0.214
Tregs	Urinary fluoride	0.088	0.021	<0.001	0.045	0.131
	IFN-γ	-0.051	0.019	0.01	-0.088	-0.013
IFN-γ	Urinary fluoride	-0.353	0.463	0.448	-1.279	0.573
	IL-37	-0.003	0.024	0.898	-0.052	0.046
	Urinary fluoride*IL-37	0.000	0.006	0.994	-0.012	0.013
Tregs	Urinary fluoride	0.086	0.023	<0.001	0.041	0.132
	IFN-γ	-0.048	0.019	0.013	-0.086	-0.010
IFN-γ	Urinary fluoride	0.150	0.207	0.470	-0.263	0.564

	TNF-α	0.634	0.101	<0.001	0.432	0.837
	Urinary fluoride*TNF-α	-0.028	0.026	0.286	-0.080	0.024
Tregs	Urinary fluoride	0.087	0.021	<0.001	0.046	0.128
	IFN-γ	-0.045	0.018	0.015	-0.082	-0.009
IFN-γ	Urinary fluoride	-0.201	0.214	0.352	-0.630	0.227
	IL-13	0.500	0.765	0.516	-1.030	2.029
	Urinary fluoride*IL-13	-0.166	0.266	0.535	-0.697	0.365
Tregs	Urinary fluoride	0.087	0.021	<0.001	0.046	0.128
	IFN-γ	-0.045	0.018	0.015	-0.082	-0.009

B, regression coefficient; SE, standard deviation; *P*: *P*-value; LLCI、ULCI, confidence interval

Adjustment: Smoke,hypertension,Cancer.

Table S7

Moderated mediation analysis of other cytokines——Model 7(IL-2).

Outcome	Predictor	B	SE	*P*	LLCI	ULCI
IL-2	Urinary fluoride	-0.088	0.094	0.356	-0.277	0.101
	IL-1β	7.944	1.551	<0.001	4.836	11.052
	Urinary fluoride*IL-1β	0.235	0.515	0.650	-0.797	1.267
Tregs	Urinary fluoride	0.081	0.022	0.001	0.036	0.126
	IL-2	-0.085	0.039	0.034	-0.163	-0.007
IL-2	Urinary fluoride	0.002	0.073	0.984	-0.146	0.149
	IL-12	0.701	0.099	<0.001	0.503	0.899
	Urinary fluoride*IL-12	0.006	0.031	0.845	-0.055	0.067
Tregs	Urinary fluoride	0.085	0.023	0.001	0.038	0.132
	IL-2	-0.087	0.041	0.037	-0.169	-0.005
IL-2	Urinary fluoride	-0.176	0.240	0.467	-0.658	0.306
	IL-37	-0.003	0.013	0.800	-0.029	0.023
	Urinary fluoride*IL-37	0.000	0.003	0.976	-0.006	0.007
Tregs	Urinary fluoride	0.082	0.024	0.001	0.034	0.131
	IL-2	-0.094	0.040	0.024	-0.175	-0.013
IL-2	Urinary fluoride	-0.010	0.095	0.914	-0.201	0.180
	IFN-γ	0.416	0.081	<0.001	0.255	0.578
	Urinary fluoride*IFN-γ	0.001	0.023	0.972	-0.046	0.048
Tregs	Urinary fluoride	0.081	0.022	0.001	0.036	0.126
	IL-2	-0.085	0.039	0.034	-0.163	-0.007
IL-2	Urinary fluoride	0.132	0.104	0.210	-0.077	0.342

	TNF-α	0.365	0.052	<0.001	0.260	0.469
	Urinary fluoride*TNF-α	-0.017	0.013	0.183	-0.042	0.008
Tregs	Urinary fluoride	0.081	0.022	0.001	0.036	0.126
	IL-2	-0.085	0.039	0.034	-0.163	-0.007
IL-2	Urinary fluoride	0.073	0.053	0.174	-0.033	0.179
	IL-4	3.767	0.323	<0.001	3.121	4.413
	Urinary fluoride*IL-4	-0.168	0.084	0.050	-0.336	0.000
Tregs	Urinary fluoride	0.081	0.022	0.001	0.036	0.126
	IL-2	-0.085	0.039	0.034	-0.163	-0.007
IL-2	Urinary fluoride	-0.199	0.115	0.090	-0.430	0.032
	IL-13	-0.130	0.405	0.749	-0.941	0.681
	Urinary fluoride*IL-13	0.047	0.141	0.742	-0.235	0.328
Tregs	Urinary fluoride	0.081	0.022	0.001	0.036	0.126
	IL-2	-0.085	0.039	0.034	-0.163	-0.007

B, regression coefficient; SE, standard deviation; *P*: *P*-value; LLCI、ULCI, confidence interval

Adjustment: Smoke,hypertension,Cancer.

Table S8.

Moderated mediation analysis of other cytokines——Model 14(IFN-γ).

Outcome	Predictor	B	SE	*P*	LLCI	ULCI
Tregs	Urinary fluoride	0.087	0.021	<0.001	0.046	0.128
	IFN-γ	-0.082	0.036	0.027	-0.154	-0.010
	IL-1β	1.047	1.520	0.494	-1.993	4.086
	Urinary fluoride*IL-1β	-0.006	0.176	0.974	-0.357	0.346
IFN-γ	Urinary fluoride	-0.307	0.136	0.027	-0.578	-0.035
Tregs	Urinary fluoride	0.073	0.022	0.002	0.029	0.117
	IFN-γ	-0.100	0.042	0.022	-0.184	-0.015
	IL-2	-0.089	0.103	0.395	-0.296	0.119
	Urinary fluoride*IL-2	0.017	0.013	0.204	-0.010	0.044
IFN-γ	Urinary fluoride	-0.388	0.138	0.007	-0.665	-0.111
Tregs	Urinary fluoride	0.091	0.021	<0.001	0.048	0.133
	IFN-γ	-0.120	0.040	0.004	-0.201	-0.039
	IL-12	-0.074	0.064	0.25	-0.202	0.054
	Urinary fluoride*IL-12	0.020	0.009	0.035	0.001	0.039
IFN-γ	Urinary fluoride	-0.348	0.141	0.016	-0.629	-0.066
Tregs	Urinary fluoride	0.087	0.020	<0.001	0.047	0.127
	IFN-γ	-0.088	0.027	0.002	-0.143	-0.034
	IL-13	-0.109	0.05	0.035	-0.209	-0.008
	Urinary fluoride*IL-13	0.04	0.019	0.039	0.002	0.078
IFN-γ	Urinary fluoride	-0.307	0.136	0.027	-0.578	-0.035
Tregs	Urinary fluoride	0.088	0.023	<0.001	0.042	0.135
	IFN-γ	-0.072	0.051	0.162	-0.173	0.030
	IL-37	0.000	0.003	0.909	-0.007	0.006

	Urinary fluoride*IL-37	0.000	0.001	0.611	-0.001	0.002
IFN-γ	Urinary fluoride	-0.344	0.149	0.025	-0.642	-0.045
Tregs	Urinary fluoride	0.093	0.021	<0.001	0.052	0.134
	IFN-γ	-0.135	0.053	0.013	-0.240	-0.030
	TNF-α	0.001	0.027	0.968	-0.053	0.055
	Urinary fluoride*TNF-α	0.007	0.005	0.172	-0.003	0.016
IFN-γ	Urinary fluoride	-0.307	0.136	0.027	-0.578	-0.035
Tregs	Urinary fluoride	0.088	0.021	<0.001	0.047	0.129
	IFN-γ	-0.086	0.043	0.050	-0.172	0.000
	IL-4	-0.199	0.295	0.503	-0.789	0.391
	Urinary fluoride*IL-4	0.056	0.044	0.204	-0.031	0.143
IFN-γ	Urinary fluoride	-0.307	0.136	0.027	-0.578	-0.035

B, regression coefficient; SE, standard deviation; *P*: *P*-value; LLCI、ULCI, confidence interval

Adjustment: Smoke,hypertension,Cancer.

Table S9.

Moderated mediation analysis of other cytokines——Model 14(IL-2).

Outcome	Predictor	B	SE	*P*	LLCI	ULCI
Tregs	Urinary fluoride	0.081	0.022	0.001	0.036	0.126
	IL-2	-0.168	0.075	0.029	-0.319	-0.018
	IL-1β	0.065	1.369	0.962	-2.680	2.810
	Urinary fluoride*IL-1β	0.160	0.252	0.530	-0.346	0.665
IL-2	Urinary fluoride	-0.170	0.072	0.022	-0.314	-0.026
Tregs	Urinary fluoride	0.084	0.024	0.001	0.036	0.131
	IL-2	-0.213	0.104	0.045	-0.421	-0.005
	IL-12	-0.111	0.086	0.203	-0.285	0.062
	Urinary fluoride*IL-12	0.04	0.019	0.041	0.002	0.077
IL-2	Urinary fluoride	-0.188	0.075	0.015	-0.338	-0.039
Tregs	Urinary fluoride	0.081	0.022	0.001	0.036	0.126
	IL-2	-0.158	0.072	0.032	-0.302	-0.015
	IL-13	-0.140	0.111	0.211	-0.363	0.082
	Urinary fluoride*IL-13	0.082	0.067	0.225	-0.052	0.216
IL-2	Urinary fluoride	-0.170	0.072	0.022	-0.314	-0.026
Tregs	Urinary fluoride	0.085	0.025	0.001	0.036	0.135
	IL-2	-0.061	0.117	0.607	-0.297	0.175
	IL-37	0.002	0.004	0.547	-0.006	0.011
	Urinary fluoride*IL-37	0.000	0.002	0.779	-0.004	0.003
IL-2	Urinary fluoride	-0.164	0.078	0.041	-0.320	-0.007
IL-2	Urinary fluoride	-0.169	0.074	0.027	-0.319	-0.020
Tregs	Urinary fluoride	0.073	0.022	0.002	0.029	0.117
	IL-2	-0.089	0.103	0.395	-0.296	0.119
	IFN-γ	-0.100	0.042	0.022	-0.184	-0.015

	Urinary fluoride*IFN-γ	0.017	0.013	0.204	-0.010	0.044
IL-2	Urinary fluoride	-0.170	0.072	0.022	-0.314	-0.026
Tregs	Urinary fluoride	0.086	0.023	<0.001	0.041	0.131
	IL-2	-0.312	0.131	0.020	-0.574	-0.050
	TNF-α	-0.013	0.031	0.670	-0.075	0.048
	Urinary fluoride*TNF-α	0.017	0.010	0.097	-0.003	0.038
IL-2	Urinary fluoride	-0.170	0.072	0.022	-0.314	-0.026
Tregs	Urinary fluoride	0.08	0.022	0.001	0.036	0.125
	IL-2	-0.127	0.135	0.354	-0.398	0.145
	IL-4	-0.532	0.367	0.153	-1.268	0.204
	Urinary fluoride*IL-4	0.127	0.083	0.130	-0.039	0.293
IL-2	Urinary fluoride	-0.170	0.072	0.022	-0.314	-0.026

B, regression coefficient; SE, standard deviation; *P: P*-value; LLCI、ULCI, confidence interval

Adjustment: Smoke,hypertension,Cancer.

Table S10.

Conditional direct effects of urinary fluoride on Tregs at values of IFN-γ， IL-2 .

Mediator variable	Moderator variable	B	SE	*P*	LLCI	ULCI
	**Model 5 [a]**					
IL-2	Low expression level of IL-37	**0.136**	**0.034**	**<0.001**	**0.068**	**0.205**
	Moderate expression level of IL-37	**0.081**	**0.024**	**0.001**	**0.033**	**0.129**
	High expression level of IL-37	0.026	0.037	0.496	-0.049	0.101
IFN-γ	Low expression level of IL-37	**0.137**	**0.031**	**<0.001**	**0.076**	**0.199**
	Moderate expression level of IL-37	**0.088**	**0.022**	**<0.001**	**0.045**	**0.131**
	High expression level of IL-37	0.039	0.034	0.250	-0.028	0.106
	**Model 14 [a]**					
IL-2	Low expression level of IL-12	0.743	-0.184	0.062	-0.377	0.010
	Moderate expression level of IL-12	2.472	-0.115	0.183	-0.287	0.056
	High expression level of IL-12	4.201	-0.047	0.589	-0.220	0.126
IFN-γ	Low expression level of IL-12	**-0.107**	**0.037**	**0.005**	**-0.180**	**-0.034**
	Moderate expression level of IL-12	**-0.072**	**0.030**	**0.021**	**-0.133**	**-0.011**
	High expression level of IL-12	-0.037	0.032	0.254	-0.101	0.027
IFN-γ	Low expression level of IL-13	**-0.085**	**0.026**	**0.002**	**-0.137**	**-0.033**
	Moderate expression level of IL-13	**-0.042**	**0.018**	**0.022**	**-0.078**	**-0.006**
	High expression level of IL-13	0.161	0.099	0.110	-0.038	0.359

P: *P*-value.

[a] B, regression coefficient; SE, standard deviation; LLCI、ULCI, confidence interval.
